# Changes in soil microbial communities after 10 years of winter wheat cultivation versus fallow in an organic-poor soil in the Loess Plateau of China

**DOI:** 10.1371/journal.pone.0184223

**Published:** 2017-09-07

**Authors:** Hui Tian, Hui Wang, Xiaoli Hui, Zhaohui Wang, Rhae A. Drijber, Jinshan Liu

**Affiliations:** 1 Key Laboratory of Plant Nutrition and Agro-environment in Northwest China, Ministry of Agriculture/College of Natural Resources and Environment, Northwest A&F University, Yangling, Shaanxi, China; 2 Department of Agronomy and Horticulture, University of Nebraska-Lincoln, Lincoln, Nebraska, United States of America; USDA Agricultural Research Service, UNITED STATES

## Abstract

Agricultural management methods, such as cultivation or fallowing, have led to significant changes in soil fertility and hence, crop yield. Such changes may have stemmed from changes in soil microbial communities and associated biogeochemical processes. This phenomenon is particularly true in organic-poor soil in the Loess Plateau of China. In this study, we examined three existing soil management regimes as part of a 10-year field experiment and evaluated their effects on fungal and bacterial community structures by performing high-throughput 454 pyrosequencing. These management regimes were (i) fertilized winter wheat (*Triticum aestivum* L.) (FW), (ii) continuous natural fallow with weeds but without crop grown (NF), and (iii) continuous bare fallow without weeds or crop grown (BF). After 10 years, soil organic carbon (SOC), microbial biomass carbon (MBC), and available potassium (K) concentrations were highest in NF. Soil N behaved differently, with BF obtaining the highest nitrate nitrogen (N). Meanwhile, slight differences in total N (TN) were observed among FW, NF, and BF. Available phosphorus (P) was highest and available K was lowest in FW. Microbial communities were dominated by Ascomycota (59.1% of fungal sequences), and Acidobacteria, Actinobacteria, Bacteroidetes, Firmicutes, and Proteobacteria (75.7% of bacterial sequences) in FW, NF and BF at the phylum level. Soil management regimes did not affect the fungal and bacterial richness and diversity but significantly modified their community compositions. Compared with FW, the abundances of Ascomycota (fungi phylum) and *Alternaria*, *Gibberella*, and *Emericella* (fungi genus) were increased by NF, whereas the values of *Chaetomium*, *Humicola*, and *Cryptococcus* (fungi genus) were decreased by BF. The abundances of Verrucomicrobia (bacteria phylum), and *Steroidobacter* (bacteria genus) were increased by NF, and *Bacteroides* (bacteria genus) was increased by BF. Canonical correspondence analysis showed that SOC, available P, and TN might be the key factors in community formation. Therefore, the decadal absence of plants (BF) affected soil fertility by increased available K and nitrate N, whileas natural fallow (NF) affected soil fertility by increased SOC, available K, and MBC, and they all changed fungal and bacterial community compositions.

## Introduction

In most unmanaged ecosystems, soil carbon (C) and nitrogen (N) cycles are closely linked by microbial processes, such as decomposition and mineralization. However, conventional agricultural practices alter these cycles, as well as the diversity and abundance of soil microorganisms [1, 2]. Consequently, the soil fertility, productivity, and diversity are changed, along with the composition of plant communities [3]. For example, N and phosphorus (P) fertilizers are applied to conventional systems to replace microbially mediated N and P mineralization, supply N and P directly to plants [4, 5], and change the biogeochemical properties of soil [6–8]. Although shifting to N and P fertilizers increases crop production, these types of fertilizers have unintended effects on soil microorganisms by changing the structure and function of microbial communities [9–13]. Fallow is the stage of crop rotation during which land is deliberately not used to grow crops. This strategy is generally used in dryland farming to conserve moisture for the main crop [14]. In an agricultural system, the management of land fallowing and the time that the land remains fallow (i.e., short summer fallow, several months as part of a two-year rotation, or abandoned) affect the amount of soil organic carbon (SOC), its fractions [15–17] and microbial community structures [18]. However, limited information is available regarding soil microbial communities in fallowed and cropped soil under the same conditions [19–21].

The Loess Plateau, which covers an area of approximately 640,000 km^2^, is located in the upper and middle reaches of the Yellow River in China. The soil in this region has a low organic C (6.15 g kg^−1^) [22] and N [23] concentration because of the low fertility of the loess parent material, but has a richness of potassium (K) nutrition. Therefore, crop (e.g. wheat) yields can be improved by using N and P fertilizers [24, 25]. In the past two decades, several cultivated lands have been transformed into grasslands or abandoned to reduce soil erosion [26, 27]. Under the management regimes long-term wheat cultivation and fallowing, the soil biogeochemical properties and microbial community of soil will change, thereby ultimately influencing present and future soil fertility. In the present work, we conducted a 10-year field experiment to study the responses of soil biogeochemical properties and microbial communities to wheat cultivation (with N and P fertilizers application) and fallowing regimes, i.e., bare fallow (means that the land is kept free of vegetation and organic amendments) versus natural fallow (means that weedy vegetation is allowed to grow naturally). For this purpose, we conducted 454 pyrosequencing that targeted the V1–V3 regions of bacterial 16S ribosomal RNA (rRNA) genes and fungal internal transcribed spacer (ITS) genes to identify and quantify soil bacterial and fungal taxa from a replicated factorial field experiment.

## Materials and methods

### Experiment site description

The field trial was conducted in the research farm of Northwest A&F University (34°17′59″N, 108°4′12″E), Yangling, Shaanxi Province, China. Prior to the experiment commenced, the land was cultivated with winter wheat by local farmers and received N and P fertilizers. This area is located on the southern Loess Plateau at an elevation of approximately 520 m on the third-level terrace of the Wei River, which is the largest tributary of the Yellow River. Monthly temperature and rainfall during the experiment are shown in S1 Fig. The average annual rainfall was 580 mm, approximately 60% of which occurred in July, August, and September. The average annual air temperature was 13°C. Soil type was classified as Eum-Orthic Anthrosol using Chinese Soil Taxonomy and the World Soil Classification of the Food and Agriculture Organization (sand 26.7%, silt 40.8%, and clay 32.5%). The soil is extremely rich in K but is deficient in P [28, 29]. In the beginning of the experiment (September 2004), the selected soil biogeochemical properties at a depth of 0 to 0.2 m were as follows: organic C, 7.96 g kg^−1^; total N, 1.07 g kg^−1^; available P (Olsen P), 15.0 mg kg^−1^; available K, 182 mg kg^−1^; nitrate N (NO_3_-N), 5.43 mg kg^−1^; pH (H_2_O), 8.25; bulk density, 1.24 g cm^−3^; electrical conductivity (EC), 135 μs cm^−1^; and CaCO_3_, 10.1%.

### Experiment design

The experiment, which was established in September 2004, consisted of three management treatments arranged in a random complete block design with three replicates. Plot size was 40 m^2^ (4 m × 10 m) with a buffer zone of 1.0 m between plots and 2.0 m between blocks. The three treatments were as follows: (i) fertilized winter wheat (FW, 160 kg N ha^−1^ year^−1^ + 43.6 kg P ha^−1^ year^−1^, i.e., the fertilization practice used by local farmers), (ii) continued natural fallow (NF) (from 2004–2014) without fertilization or wheat cultivation, and (iii) continued bare fallow (BF) (from 2004–2014) without fertilization or wheat cultivation. Urea (containing 46% N) was used as the N fertilizer, and single superphosphate (2004–2008, containing P 7.0%) and triple superphosphate (2009–2014, containing P 20% and S 1.5%) was used as the P fertilizer. Both were broadcast on the soil surface and incorporated into the soil at a depth of 10 cm via rotary tilling as basal fertilizers prior to wheat sowing. Winter wheat (*Triticum aestivum* L. cv. Xiaoyan 22) was sown using a seeder machine at 135 kg seed ha^−1^ with a row spacing of 20 cm in early October and was harvested in early June of the following year. The wheat was grown under rainfed conditions and was the only crop grown each year. Wheat straw was returned to the soil, and weeds were manually removed. The period after harvesting winter wheat and before sowing the next cycle of winter wheat is the summer fallow season. During this period, the fields were tilled by deep plowing one week after wheat harvest followed by rotary tilling one week before wheat sowing. No human disturbance or intervention, including fertilization and tillage, occurred in NF, thereby allowing the vegetation to grow naturally throughout the experiment period (2004–2014). Vegetation had nearly 100% coverage and was dominated by Leguminosae and Gramineae in spring and summer; however, minimal green vegetation (Gramineae) was found to grow in winter because of the low temperatures (the lowest recorded temperature during the experiment period was –14°C). In BF, soil was kept bare by manually removing vegetation. Similar to FW, tillage was also used in BF.

### Soil sampling and preparation

On June 10, 2014 (after wheat harvest), soil samples were randomly collected from 4 points in each plot at 0–20 cm depth by using a 5.0-cm-diameter auger. Soil from the 4 cores in a plot was mixed to obtain 1 composite sample. Soil samples were passed through a 2-mm sieve and divided into two subsamples: ones were air-dried for analysis of soil physical-chemical properties, and the other subsamples were immediately transported on ice to the laboratory and stored at 4°C until needed analysis of soil microbial biomass C and N (MBC and MBN) and inorganic N (NO_3_−N and NH_4_−N), or at −80°C prior to genomic DNA extraction.

### DNA extraction and pyrosequencing analysis

DNA was extracted from the soil samples (0.5 g) using an E.Z.N.A.^™^ Soil DNA Kit (Omega Bio-Tek Inc., Norcross, GA, USA) in accordance with the protocol of the manufacturer. The quantity and quality of the DNA extracts were determined using a NanoDrop 1000 spectrophotometer (Thermo Fisher Scientific Inc., Waltham, MA, USA). The extracted DNA was stored at −20°C prior to further analyses.

An aliquot of the extracted DNA from each sample was used as the template for amplification. The V1–V3 hypervariable regions of the bacterial 16S rRNA gene sequences and the ITS region of the fungal rRNA gene sequences were amplified. Amplicon libraries were prepared using tagged bacterial and fungal universal primers, i.e., 8F and 533R for bacteria, and ITS5 and ITS4 for fungi. The DNA samples were amplified individually using the fusion primer pairs 27F (5′-*A*-MID- AGAGTTTGATCCTGGCTCAG-3′) and 533R (5′-*B*-TTACCGCGGCTGCTGGCAC-3′) [30] for bacteria, and ITS5 (5′-*A*-MID- GGAAGTAAAAGTCGTAACAAGG-3′) and ITS4 (5′-*B*-TCCTCCGCTTATTGATATGC-3′) [31] for fungi to generate polymerase chain reaction (PCR) fragments, where *A* and *B* denote the two pyrosequencing primers (20 bp, 454 Life Sciences, 15 Commercial Street Branford, CT, USA) and MID denotes the multiplexing barcode tags (7 bp) for post-sequencing reading. The PCR reactions were performed in a 25 μL mixture containing 1.0 μL of each primer at 10 μM, 2.0 μL template DNA (20 ng/μL), 2.5 μL of 10× buffer, 0.125 μL of Pyrobest DNA Polymerase (5 U/μL, Takara Bio, Dalian, China), 2.0 μL of deoxy-ribonucleoside triphosphate (dNTP) at 2.5 mM, and 16.375 μL of ultrapure sterile water. The following thermal program was used for amplification: 94°C for 4 min, followed by 27 cycles of denaturation at 94°C for 30 s, annealing at 55°C (bacteria) [32] or 47°C (fungi) for 45 s [33], extension at 72°C for 1 min, and a final extension at 72°C for 7 min. The PCR products were purified using AMPure XP beads (Agencourt, Beckman Coulter, Beverly, MA, USA) and quantified using a Quant-iT^™^ PicoGreen^®^ dsDNA Assay Kit (Invitrogen Life Technologies, Carlsbad, CA, USA) in accordance with the instructions of the manufacturer. Thereafter, the products of the amplicons were pooled at equimolar concentrations to build the sequencing library and sequenced using a Roche 454 GS FLX+ pyrosequencing machine (Roche, 454 Life Sciences, Branford, CT, USA) in accordance with the instructions of the manufacturer at the Personal Biotechnology Co., Ltd., Shanghai, China.

### Sequences processing and analysis

After 454 pyrosequencing steps were conducted, downstream sequence analyses were performed using the Quantitative Insights into Microbial Ecology (QIIME, version 1.7.0) [34] and UChime [35] of Mothur (version 1.31.2) [36] before further statistical analyses were conducted. The sequence reads were split by identifying the barcodes, and quality filters were applied to remove low-quality reads. Filters that fell under the following criteria were eliminated: (i) sequences with less than 200 bp, (ii) sequences with quality scores lower than 25, (iii) sequences that contained more than six ambiguous or homologous sequences (default parameters), (iv) sequences with a maximum primer mismatch greater than 1, and (v) chimera sequences detected by UChime. All high-quality sequences (including the singletons) with a distance value below 0.03 were grouped into operational taxonomic units (OTUs) using QIIME’s UClust [37]. Furthermore, taxonomic assignment of the sequences was conducted for each sample by using the QIIME’s BLAST (with an *E* value of 10^−3^ by default parameters) [38] based on the Greengenes [39] (Release 13.8, http://greengenes.secondgenome.com/) and Unite databases (Release 5.0 http://unite.ut.ee/index.php) [40] for bacteria and fungi, respectively. The sequences were deposited into the Sequence Read Archive database of the National Center for Biotechnology Information (accession number: SRP087715). Three metrics (the observed OTU richness, the Chao1 index, and the Shannon index) were used to access the microbial alpha diversity by the Mothur software (version 1.31.2). The microbial communities were compared by performing principal coordinates analysis (PCoA) of the Fast UniFrac (http://bmf.colorado.edu/unifrac) metric matrix based on phylogenetic information [41]. Weighted Fast UniFrac distances between the samples were calculated, and PCoA was conducted on the distance measured, and the coordinates were used to draw the 2D graphical outputs.

### Soil analytical methods

Soil pH was measured in water (1:2.5 soil/water) by a pH meter (PHS-3C, INESA Scientific Instrument Co., Ltd, China). Available P (Olsen P) were extracted with 50 mL of 0.5 mol L^−1^ NaHCO_3_ (pH 8.5) [42] and determined using an injection pump analyzer (AA3, Bran + Luebbe, Germany). Available K was extracted with 1 mol L^−1^ NH_4_OAc and determined by a photoelectric flame photometer [43, 44]. Soil EC was measured in water (1:5 soil/water) by an EC meter (DDS-307A, INESA Scientific Instrument Co., Ltd, China). Soil bulk density was measured on the 100 cm^3^ undisturbed soil cores. Soil organic carbon was determined using a wet oxidation procedure with potassium dichromate (K_2_Cr_2_O_7_)-sulfuric acid (H_2_SO_4_) [45, 46]. Soil total N was determined by Kjeldahl method [47]. Inorganic N (nitrate N (NO_3_−N) and ammonium N (NH_4_−N)) were extracted with 1 mol L^−1^ potassium chloride using field-moist soil (2-mm sieve), and it was determined with an injection pump analyzer (AA3, Bran + Luebbe, Germany). Soil microbial biomass C (MBC) and N (MBN) were measured by chloroform fumigation–extraction method [48, 49]. Organic C in the filtrate was determined with a total organic carbon analyzer (Shimadzu TOC-V_CPH_) [50]. Total N in the filtrate was treated by alkaline persulfate oxidation and measured with dual-wavelength ultraviolet spectrophotometry [51]. A *K*_C_ of 0.45 and *K*_N_ of 0.54 were used to convert the differences between organic C and N extracted from chloroform-fumigated and unfumigated soil samples into MBC and MBN, respectively [50].

### Statistical analysis

The one-factorial ANOVA procedure in SAS (v8.0, SAS Institute Inc., USA) was used to perform data analysis on soil biogeochemical properties and alpha diversity. The least significant difference (LSD) test at *P* < 0.05 was adopted to assess the differences in the means of three replicates for soil management treatments. Data on the differences in fungal and bacterial community compositions among the four management treatments was were obtained with the metastats [52] command of the mothur software (version 1.31.2) and *P* values of < 0.05 were considered significant. To test for significant differences in soil community compositions among the different treatments, an analysis of similarities (ANOSIM) was also conducted using QIIME based on Bray-Curtis distance measures (unweighted Fast UniFrac distances) and abundance data. Canonical correspondence analysis (CCA) [53] was conducted using the Vegan package [54] for R (https://www.r-project.org/) to analyze the relationships between the community structures (OTU profiles) of the different samples and soil physiochemical properties.

## Results

### Soil biogeochemical properties

The 10-year soil management regimes significantly changed the concentrations of SOC, available P, available K, NO_3_−N, and microbial biomass carbon (MBC). However, they did not alter soil total N (TN), microbial biomass N (MBN), pH, EC, and bulk density (Table 1). Compared with NF treatment, the concentrations of SOC, available K, and MBC were decreased by FW, and BF treatments. By contrast, soil available P concentration was increased by FW treatment and NO_3_−N concentration was increased by BF treatment.

**Table 1:** Biogeochemical properties of the soil samples under different soil management regimes.

Soil properties	Treatments		
	FW	NF	BF
Soil organic C (SOC) (g kg^−1^)	9.55 (0.12) b	10.76 (0.37) a	9.44 (0.16) b
Total N (TN) (g kg^−1^)	1.04 (0.03) a	0.96 (0.06) a	1.03 (0.03) a
pH (soil: water = 1: 2.5)	8.28 (0.01) a	8.26 (0.04) a	8.33 (0.04) a
Available P (Olsen P) (mg kg^−1^)	15.91 (1.32) a	7.07 (0.59) b	5.69 (0.23) b
Available K (mg kg^−1^)	133.5 (8.26) c	185.8 (2.32) a	166.7 (8.34) b
NO_3_−N (mg kg^−1^)	9.61 (0.68) b	8.04 (0.79) b	12.73 (1.31) a
NH_4_−N (mg kg^−1^)	n.d.^#^	n.d.	n.d.
Microbial biomass C (MBC) (mg kg^−1^)	95.55 (1.80) b	159.14 (13.9) a	90.09 (4.12) b
Microbial biomass N (MBN) (mg kg^−1^)	21.56 (3.08) a	23.03 (1.69) a	25.80 (2.77) a
EC (μs cm^−1^)	87.4 (2.95) a	95.4 (5.29) a	90.0 (2.71) a
Bulk density (g cm^−3^)	1.29 (0.02) a	1.31 (0.05) a	1.36 (0.01) a

^#^n.d., not detected.

Values in the brackets represent the standard error (n = 3). Different letters in a row indicate differences between treatments (ANOVA followed by LSD *post hoc* test, *P* < 0.05, n = 3). FW, fertilized wheat; NF, natural fallow; BF, bare fallow.

### Richness and diversity of fungi and bacteria

A total of 146,678 ITS and 255,167 16S rRNA gene sequence reads were generated by pyrosequencing 9 samples from the three management treatments. After filtration, the total number of high-quality sequences was 114,161 in the fungal sequence library and 214,289 in the bacterial sequence library. The sequence number of each treatment are detailed in S1 Table.

The OTU number, Chao 1, and Shannon indices are summarized in Table 2. For fungi and bacteria, the OTU number and the Chao 1 and Shannon indices did not considerably differ (*P* > 0.05) among FW, NF, and BF. Similar trends were observed among the plots of OTU number versus sequence number (i.e., rarefaction curves), as shown in S2 Fig.

**Table 2:** Richness and diversity of fungal and bacterial gene fragment sequences in the soil samples under the three management treatment regimes.

	FW	NF	BF
	fungi		
OTU number	465 (27) a	464 (50) a	368 (45) a
Chao 1	560 (55) a	648 (76) a	552 (66) a
Shannon	4.37 (0.05) a	3.43 (0.57) a	3.89 (0.39) a
	bacteria		
OTU number	3171 (104) a	3585 (196) a	3538 (229) a
Chao 1	4790 (128) a	5752 (1465) a	5623 (400) a
Shannon	7.08 (0.42) a	6.91 (1.34) a	6.11 (0.29) a

Values in the brackets represent the standard error (n = 3). Different letters in a row indicate differences between treatments (ANOVA followed by LSD *post hoc* test, *P* < 0.05). FW, fertilized wheat; NF, natural fallow; BF, bare fallow.

### Fungal community structure

**Fig 1:**
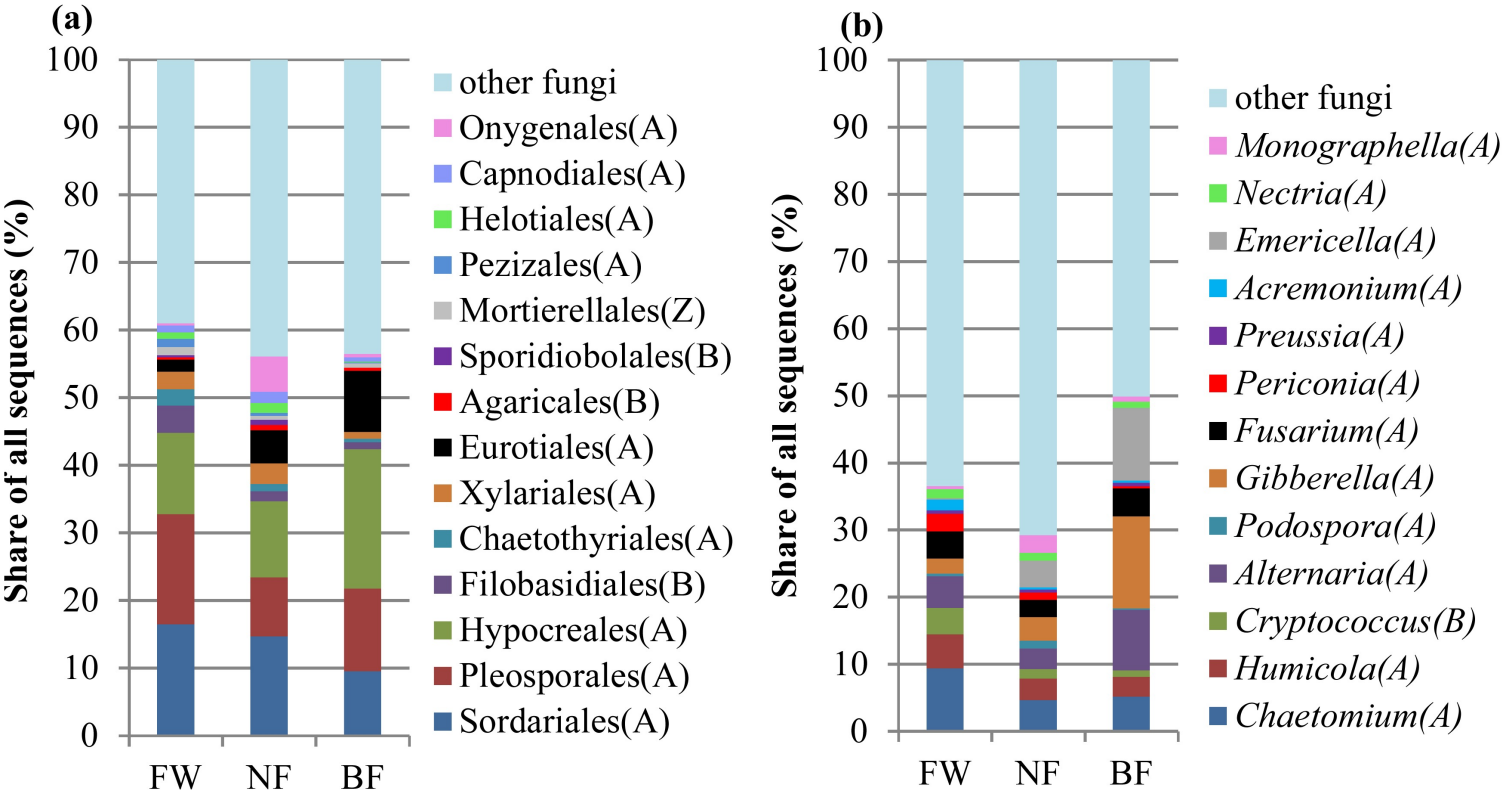
Relative abundances of major taxonomic groups (with abundance > 1% in at least one treatment, only identified sequences classified under a specific taxon were considered) at the (a) order and (b) genus levels across the three soil management regimes. The data represent the mean values of the three replications. The postfix means the taxon is belong to a special phylum. A, Ascomycota; B, Basidiomycota; Z, Zygomycota. FW, fertilized wheat; NF, natural fallow; BF, bare fallow.

The ITS gene sequences were affiliated with 5 phyla, 69 orders, and 297 genera (except for the unidentified sequences assigned to the Kingdom Fungi). The dominant fungal phyla (S3 Fig) and orders (Fig 1a) in all the soil samples were Ascomycota (59.1% of fungal sequences) and Sordariales, Pleosporales, Hypocreales, Filobasidiales, and Chaetothyriales (44.1% of fungal sequences). Although ANOSIM (S2 Table) indicated that the fungal community structures did not differ among some of the treatments, the community compositions exhibited several differences (Fig 1 and S3 Table). The relative abundance of Ascomycota in FW and NF (with an average value of 53.2%) was considerably lower (S3 Table, *P* < 0.05) than that in BF (71.0%). However, no significant difference was observed among FW and NF at the fungal order levels. The relative abundances of genera *Chaetomium* (5.1%), *Humicola* (2.9%), and *Cryptococcus* (0.9%) in BF were less (*P* < 0.05) than those in FW (with values of 9.4%, 5.1%, and 3.9%, respectively) (Fig 1b). The relative abundances of genera *Alternaria* (9.0%), *Gibberella* (13.7%), and *Emericella* (10.8%) in BF were greater (*P* < 0.05) than those in FW or NF (average values of 3.9%, 2.8%, and 2.0%, respectively).

### Bacterial community structure

**Fig 2:**
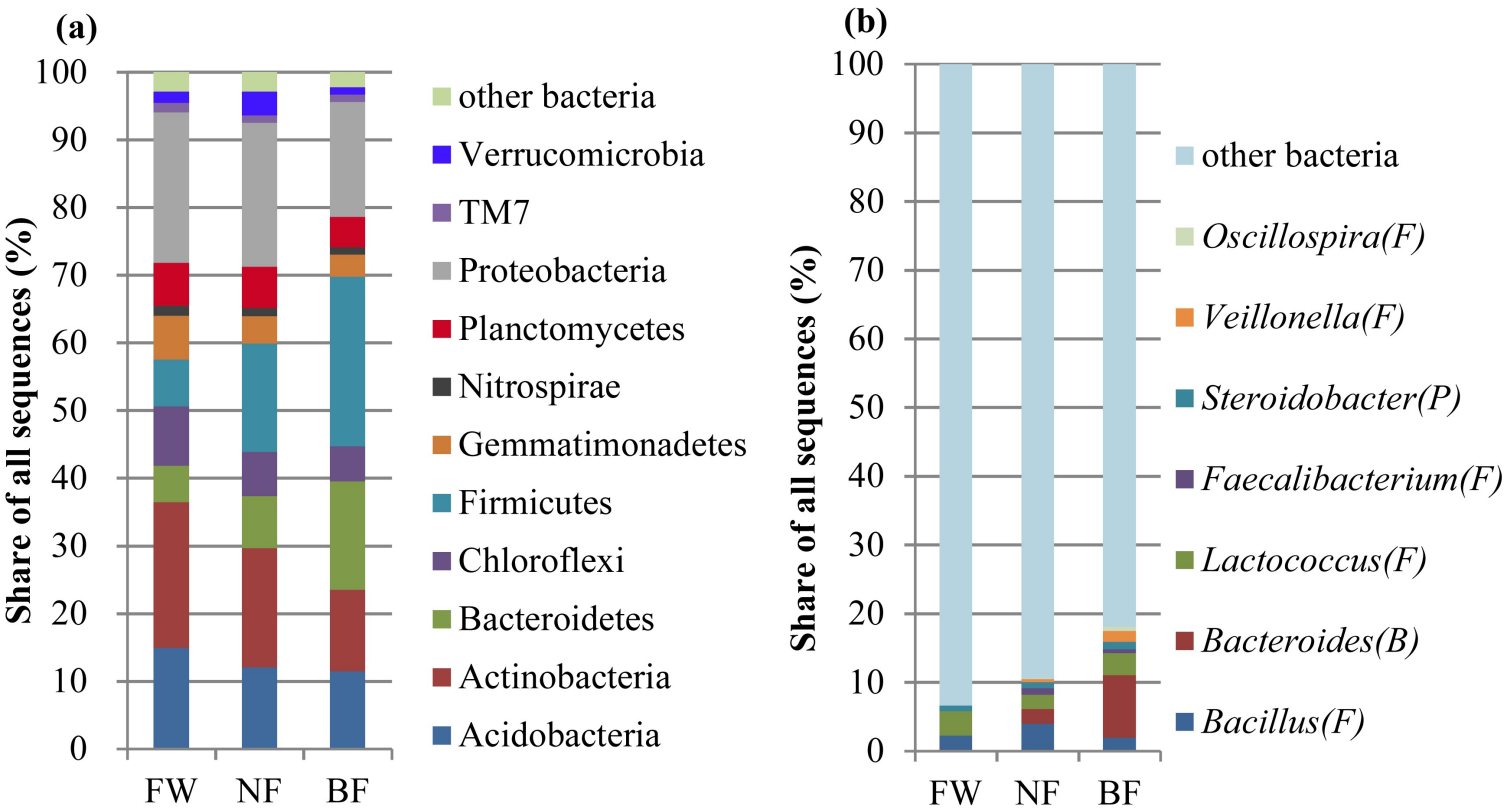
The relative abundance of major taxonomic groups (with abundance > 1% in at least one treatment, only identified sequences classified under a specific taxon were considered) at the (a) phylum and (b) genus levels across the three soil management regimes. The data represent the mean values of the three replications. The postfix means the taxon is belong to a special phylum. A, Actinobacteria; B, Bacteroidetes; F, Firmicutes; N, Nitrospirae; P, Proteobacteria. FW, fertilized wheat; NF, natural fallow; BF, bare fallow.

The 16S rRNA gene sequences were affiliated with 37 phyla and 746 genera. The dominant phyla in all the soil samples were Acidobacteria, Actinobacteria, Bacteroidetes, Firmicutes, and Proteobacteria, which represented an average of 82.5% of all the bacterial sequences (Fig 2a). ANOSIM (S2 Table) showed that the bacterial community structure did not differ among the three treatments, however the statistical analysis (S4 Table) showed that the relative bacterial abundance of Verrucomicrobia differed significantly between FW and NF, and NF and BF at the phylum level. At the genus level (Fig 2b), the dominant bacterial genera in all the soil samples were *Bacillus*, *Bacteroides*, *Lactococcus*, and *Steroidobacter*, which represented an average of 10.3% of all the bacterial sequences. *Bacteroides* (9.1%) was the most dominant genera in BF, but significantly decreased (*P* < 0.05) in FW and NF (with the values of 0.01% and 2.2%, respectively). No significant differences in the relative abundances of other dominant genera (with > 1%) were observed among the three treatments (Fig 2b), whileas the relative abundances of the other genera (with <1%) (e.g. *Steroidobacter*, *Streptomyces*, *Pirellula*, etc.) exhibited differences between FW and NF, and NF and BF (data not shown).

### Comparison of fungal and bacterial communities among treatments and their relationship with soil biogeochemical properties

**Fig 3:**
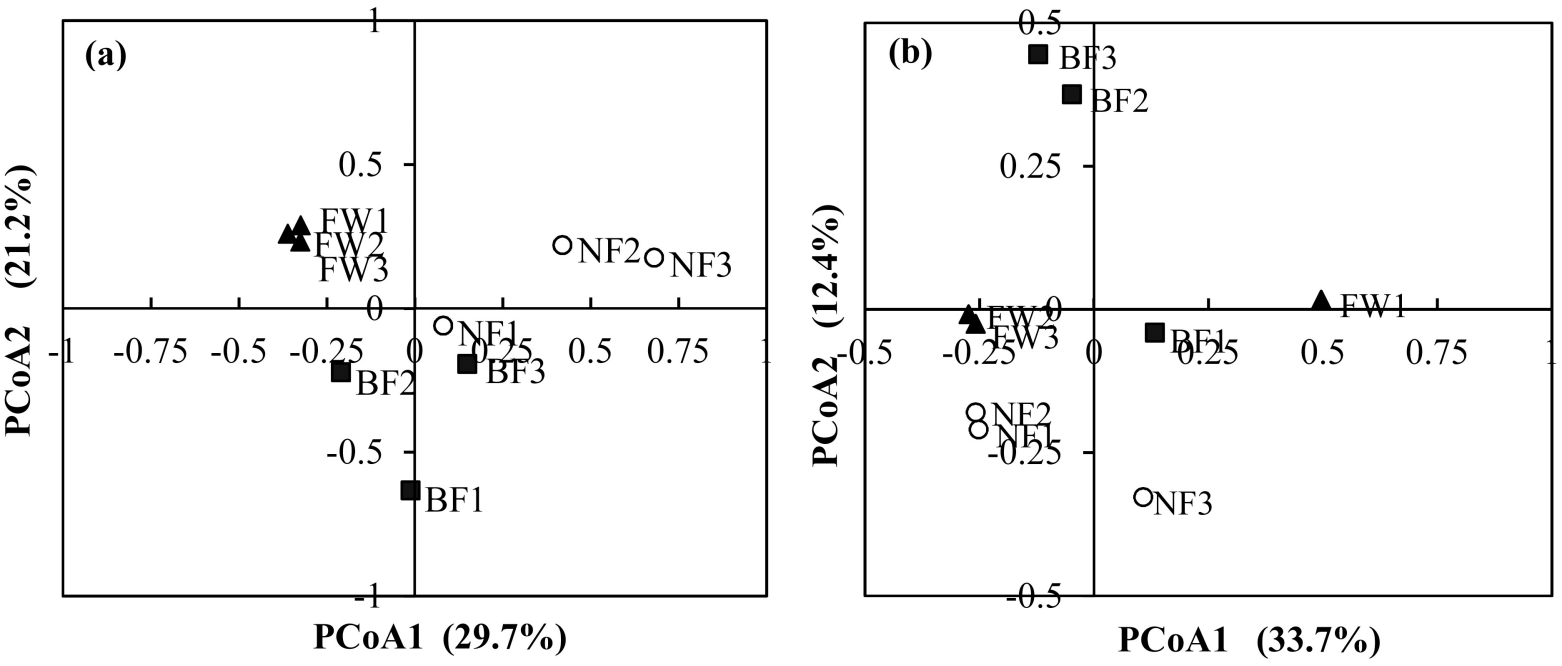
PCoA of the dissimilarity between the treatments for (a) fungi and (b) bacteria. PCoA of the OTU matrix obtained from soil samples subjected to different management regimes based on the weighted Fast UniFrac metric. The first two axes are drawn and the percent of variance explained by each axis is provided. FW, fertilized wheat; NF, natural fallow; BF, bare fallow.

The first two principal coordinates represented 50.9% of the variation in fungal (Fig 3a) and 46.1% of the variation in bacterial (Fig 3b) communities according to the principal coordinates analysis (PCoA). The fungal community structures of FW differed from those of NF and BF treatments (Fig 3a), whereas, the bacterial community structures of NF were distinct from the FW and BF treatments (Fig 3b).

**Fig 4:**
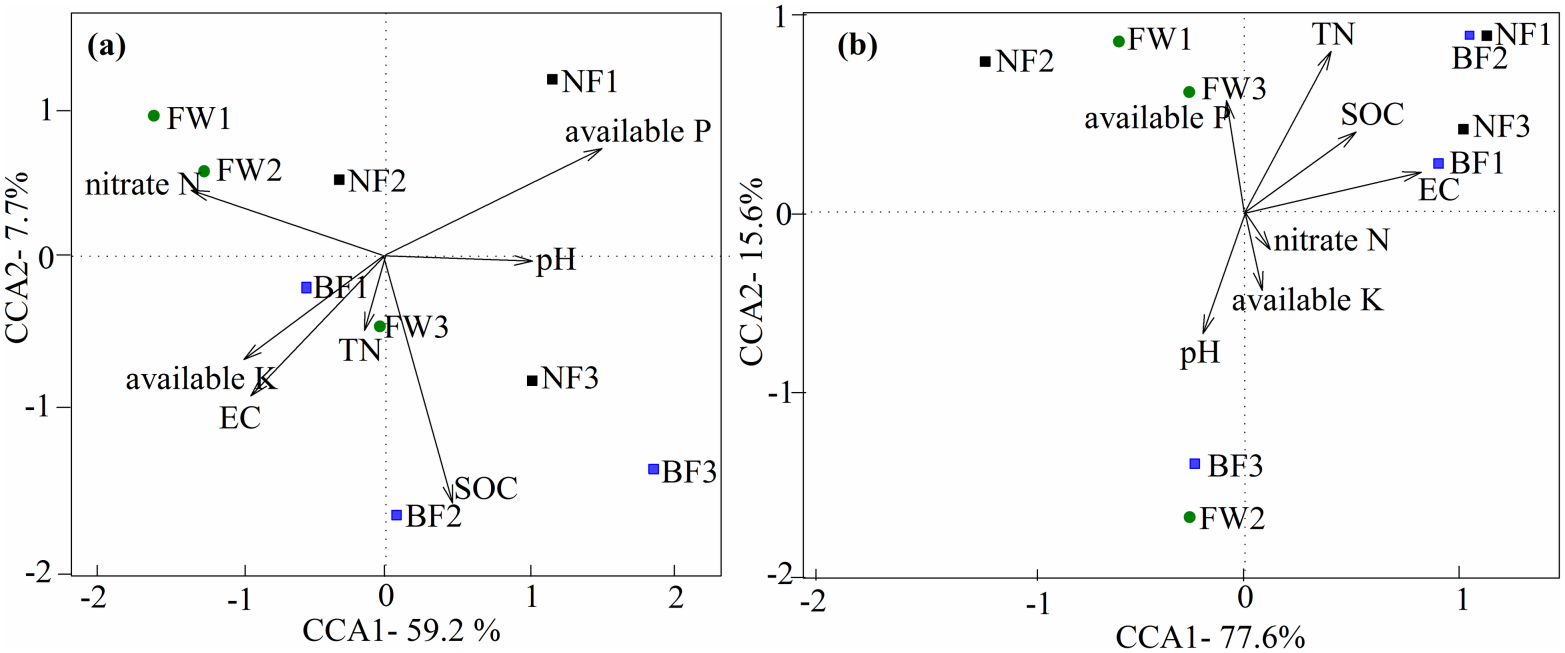
CCA performed on the samples under different management regimes using (a) fungal and (b) bacterial OTU profiles and soil biogeochemical properties. The direction of arrows indicates correlation with the first two canonical axes and the length of arrows represents the strength of the correlations. SOC, soil organic carbon; TN, soil total N; EC, electrical conductivity. FW, fertilized wheat; NF, natural fallow; BF, bare fallow.

The CCA results indicated that 66.9% and 93.2% of the total variance in fungal (Fig 4a) and bacterial (Fig 4b) community structures could be explained by the first and second axes (CCA1 and CCA2), respectively. For fungi (Fig 4a), nitrate N and available P exhibited negative and positive relationships, respectively, to axis CCA1, whereas SOC and EC demonstrated negative relationships to axis CCA2. For bacteria (Fig 4b), SOC and EC presented positive relationships to axis CCA1. By contrast, TN and available P exhibited a positive relationship to axis CCA2, and pH presented a negative relationship to axis CCA2. However, none of all the analyzed environmental factors were found to have strongly influenced soil fungal and bacterial communities, as indicated in the Monte Carlo permutation test.

## Discussion

### Effects of long-term wheat cultivation on soil biogeochemical properties and microbial communities

Soil amendment with fertilizers can improve plant growth and health, and in turn, enhance soil biogeochemical properties. A 20-year field experiment conducted in northwestern China showed that MBC concentration in FW was significantly lower than that in NF during winter wheat harvest, whileas no significant difference in SOC and TN concentrations was observed between FW and NF [15]. In the present study, similar trends were observed for TN concentration (Table 1). However, significant difference was observed between FW and NF in terms of SOC and MBC concentrations (Table 1), which agree with the 22-year field experiment that conducted in southern China, where the MBC, MBN, and SOC concentrations in FW were all significantly lower than those in NF [55]. In addition, FW increased soil available P compared with NF and BF treatments, which agree with the result of a previous long-term field experiments in southern [55] and northwestern China [56]. Several previous studies have also revealed that FW increases the soil TN, available P, MBC, and SOC compared with the treatment of wheat cultivation with no fertilizers (CK) [15, 55]. These results indicated that the wheat cultivation with fertilizers can improve the chemical and biological fertility of the soils with low SOC.

Soil amendment with fertilizers can modify the structure and/or biological activity of soil microbial communities [11, 55, 57, 58]. In the present study, FW did not alter fungal and bacterial richness and diversity compared with NF (Table 2). This finding does not correlate with the results reported by of Xue et al. [55] in red soil (Ferralic Cambisol) in southeastern China, where they found that the bacterial OTU number and Shannon diversity index were lower for fertilized treatment than for the natural fallow plots. This result suggested that the long-term application of N and P fertilizers to low-organic carbon soil with low levels of N and P nutrition did not alter microbial diversity, and the response of microbial diversity to N and P enrichment may be site- or soil-specific.

In the present study, FW did not alter the relative abundances of fungal phyla (S1 Fig). However, FW changed the abundances of certain fungal genera compared with NF (Fig 1). Similar results were also obtained in a loamy textured Cambisol (cropland) in Lusignan, France [59], where wheat cultivation with long-term fertilization altered the relative abundances of *Gemmatimonadete*, *Chloroflexi*, *Nitrospirae*, and *Ascomycota* compared with the grassland (close to the cropland, separated only by a 5 m pathway). With regard to the bacteria, FW did not change the abundances of most phyla compared with NF (Fig 2). However, in a red soil in southern China, there were significantly difference in the abundances of Proteobacteria, Firmicutes, Acidobacteria, and Actinobacteria between fertilized treatment (NP) and NF (fallow) treatment [55]. The different effects of fertilization practices on soil bacterial community compositions can be attributed to different soil biogeochemical properties, such as SOC levels with the values of 9.55 g kg^−1^ (FW) and 10.76 g kg^−1^ (NF) in the present study versus 9.51 g kg^−1^ (NP) and 11.95 g kg^−1^ (fallow) in southern China [55], and climate (annual temperature of 13°C and a mean annual precipitation of 580 mm in the present study versus annual temperature of 18°C and a mean annual precipitation of 1296 mm in southern China).

### Effects of long-term fallow management on biogeochemical properties and microbial communities

Fallow management, including BF and NF, affects soil biogeochemical properties. For example, BF significantly reduced SOC concentrations at a soil depth of 0–7.5 cm compared with no fallow in a 17-year field experiment in Brazil [17] and decreased total SOC concentrations compared with the initial values in six long-term (>30 years) BF experiments conducted in Europe [16]. By contrast, management using NF increased SOC and MBC concentrations compared with cropping in certain soil types [15]. Similar trends were observed in SOC, available K, and MBC when comparing NF with BF or FW (Table 1) in the present study. However, TN concentrations did not differ among FW, NF, and BF (Table 1), which contradicts the findings of Fan and Hao [56], who have reported that there was significant difference in TN concentration between BF and FW in a 16-year field experiment in northwestern China. These finding suggest that NF can improve soil fertility compared with BF, which can be attributed to the lack of tillage and input of organic materials in this management regime.

In the present study, BF did not affect the fungal and bacterial diversities compared with FW (Table 2). This finding is consistent with that of Guenet et al. [20], who have reported that arable and BF soils do not differ in the Shannon index. In addition, NF had no effect on fungal and bacterial diversities, which are consistent with previous reports in the Bolivian highlands [18], where the overall diversities of fungi and bacteria did not differ between NF (thola) and BF (non-thola) soils. However, 4 phyla, 24 orders, 17 genera of fungi and 13 phyla, and 55 genera of bacteria differed significantly between NF and BF after long-term (20 or 30 years) fallowing. In the present study, only 1 phylum, 1 order and 3 genera of fungi (Fig 1 and S3 Table) and 1 phylum and 7 genera of bacteria (Fig 2 and S4 Table) differed significantly between NF and BF. This difference between these studies may be attributed to the different land use history and soil biogeochemical properties (Table 1 in the present study and Table 1 in Gomez-Montano et al. [18]). In the present study, the land was continuously cultivated for crops before the current experiment was initiated. In the study of Gomez-Montano et al. [18], the land was diversely farmed with a few years of crop production, typically potato, followed by quinoa (*Chenopodium quinoa*) and barley (*Hordeum vulgare* L.), and then fallowed.

## Conclusions

In this study, soil management regimes, including wheat cultivation and fallowing, resulted in changes in soil biogeochemical properties, with the increases of SOC and MBC in NF and nitrate N in BF, and the decreases of available P in NF and BF. Consequently, the management regimes affected soil fungal and bacterial community compositions but did not differ the fungal and bacterial richness and diversity. Furthermore, canonical correspondence analysis showed that SOC, available P, and TN might be the key factors in community formation, thereby suggesting that soil biogeochemical properties influenced soil microbial community compositions. These factors are linked to the management regimes of soil in the Loess Plateau of China.

## Supporting information

S1 Fig Maximum, minimum, and mean monthly temperature (T), and monthly rainfall during the experiment (2004–2014). (DOCX)

S2 Fig Rarefaction curves for (a) fungi and (b) bacteria in the three soil management regimes. (DOCX)

S3 Fig Relative abundances of major taxonomic groups at the phylum level across the three soil management regimes. (DOCX)

S1 Table Sequence reads were generated by the pyrosequencing of 9 samples from the three soil management regimes. (DOCX)

S2 Table ANOSIM on fungal and bacterial communities among the three soil management regimes. (DOCX)

S3 Table Difference in fungal abundance at the phylum level among the three soil management regimes. (DOCX)

S4 Table Difference in bacterial abundance at the phylum level among the three soil management regimes. (DOCX)
