# Supplementary material for: Changes in soil microbial communities after 10 years of winter wheat cultivation versus fallow in an organic-poor soil in the Loess Plateau of China
Source: PLoS One. 2017 Sep 7;12(9):e0184223. doi: 10.1371/journal.pone.0184223 (PMC5589179; doi:10.1371/journal.pone.0184223)
Supplement: S1 Fig — (DOCX) [file pone.0184223.s001.docx]

**S1 Fig.** Maximum, minimum, and mean monthly temperature (T), and monthly rainfall during the experiment (2004–2014).
