# Supplementary material for: Changes in soil microbial communities after 10 years of winter wheat cultivation versus fallow in an organic-poor soil in the Loess Plateau of China
Source: PLoS One. 2017 Sep 7;12(9):e0184223. doi: 10.1371/journal.pone.0184223 (PMC5589179; doi:10.1371/journal.pone.0184223)
Supplement: S3 Fig — (DOCX) [file pone.0184223.s003.docx]

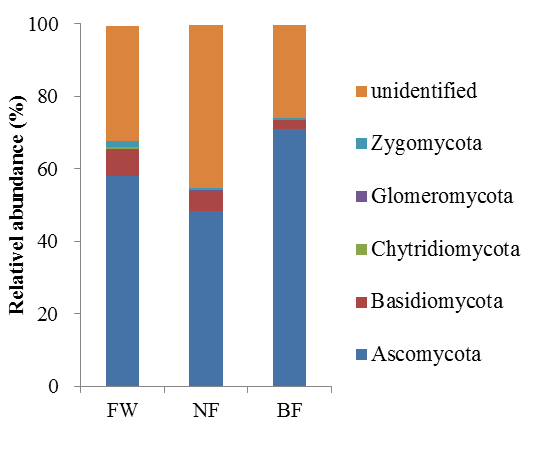


**S3 Fig.** Relative abundances of major taxonomic groups at the phylum level across the three soil management regimes. FW, fertilized wheat; NF, natural fallow; BF, bare fallow.
