# Supplementary material for: Changes in soil microbial communities after 10 years of winter wheat cultivation versus fallow in an organic-poor soil in the Loess Plateau of China
Source: PLoS One. 2017 Sep 7;12(9):e0184223. doi: 10.1371/journal.pone.0184223 (PMC5589179; doi:10.1371/journal.pone.0184223)
Supplement: S1 Table — (DOCX) [file pone.0184223.s004.docx]

**S1 Table.** Sequence reads were generated by the pyrosequencing of 9 samples from the three soil management regimes.

| Treatments | All valid sequences^#^ | High quality sequences | Percent (%) |
| --- | --- | --- | --- |
|  | fungi | | |
| FW | 16,480 | 12,438 | 75.4 |
| NF | 15,801 | 11,979 | 76.0 |
| BF | 16,612 | 13,637 | 81.4 |
|  | bacteria | | |
| FW | 19,246 | 15,884 | 80.2 |
| NF | 28,317 | 24,206 | 85.7 |
| BF | 37,492 | 31,340 | 83.3 |

#, the value in the table was the mean of the three replications. FW, fertilized wheat; NF, natural fallow; BF, bare fallow.
