# Supplementary material for: Changes in soil microbial communities after 10 years of winter wheat cultivation versus fallow in an organic-poor soil in the Loess Plateau of China
Source: PLoS One. 2017 Sep 7;12(9):e0184223. doi: 10.1371/journal.pone.0184223 (PMC5589179; doi:10.1371/journal.pone.0184223)
Supplement: S2 Table — (DOCX) [file pone.0184223.s005.docx]

**S2 Table.** ANOSIM on fungal and bacterial communities among the three soil management regimes.

| Between treatments | Fungi | | Bacteria | |
| --- | --- | --- | --- | --- |
|  | R statistic | *P* values | R statistic | *P* values |
| FW-BF | 0.48 | 0.21 | 0.04 | 0.49 |
| FW-NF | 0.59 | 0.10 | 0.26 | 0.20 |
| NF-BF | 0.44 | 0.17 | 0.52 | 0.10 |

ANOSIM, Analysis of Similarity. R = degree of separation between test groups ranging from −1 to 1; R = 0, not different; R = 1, completely different; p values were based on 999 permutations. FW, fertilized wheat; NF, natural fallow; BF, bare fallow.
