# Supplementary material for: Changes in soil microbial communities after 10 years of winter wheat cultivation versus fallow in an organic-poor soil in the Loess Plateau of China
Source: PLoS One. 2017 Sep 7;12(9):e0184223. doi: 10.1371/journal.pone.0184223 (PMC5589179; doi:10.1371/journal.pone.0184223)
Supplement: S3 Table — (DOCX) [file pone.0184223.s006.docx]

**S3 Table.** Difference in fungal abundance at the phylum level among the three soil management regimes.

| **Phylum** | **Group** | **Group** | **Sig.** | **Phylum** | **Group** | **Group** | **Sig.** |
| --- | --- | --- | --- | --- | --- | --- | --- |
| Ascomycota | BF | FW | 0.042* | Basidiomycota | BF | FW | 0.078 |
|  |  | NF | 0.047* |  |  | NF | 0.097 |
|  | FW | BF | 0.042* |  | FW | BF | 0.078 |
|  |  | NF | 0.761 |  |  | NF | 0.749 |
|  | NF | BF | 0.047* |  | NF | BF | 0.097 |
|  |  | FW | 0.761 |  |  | FW | 0.749 |
| Chytridiomycota | BF | FW | 0.126 | Glomeromycota | BF | FW | 0.477 |
|  |  | NF | 0.214 |  |  | NF | 0.194 |
|  | FW | BF | 0.126 |  | FW | BF | 0.477 |
|  |  | NF | 0.216 |  |  | NF | 0.316 |
|  | NF | BF | 0.214 |  | NF | BF | 0.194 |
|  |  | FW | 0.216 |  |  | FW | 0.316 |
| Zygomycota | BF | FW | 0.111 |  |  |  |  |
|  |  | NF | 0.336 |  |  |  |  |
|  | FW | BF | 0.111 |  |  |  |  |
|  |  | NF | 0.201 |  |  |  |  |
|  | NF | BF | 0.336 |  |  |  |  |
|  |  | FW | 0.201 |  |  |  |  |

Sig., significance. The difference in community compositions among the four management treatments was carried out with the metastats (http://metastats.cbcb.umd.edu) command of the mothur software (version 1.31.2) and *P* values of < 0.05 were considered signiﬁcant (*). FW, fertilized wheat; NF, natural fallow; BF, bare fallow.
