# Supplementary material for: Changes in soil microbial communities after 10 years of winter wheat cultivation versus fallow in an organic-poor soil in the Loess Plateau of China
Source: PLoS One. 2017 Sep 7;12(9):e0184223. doi: 10.1371/journal.pone.0184223 (PMC5589179; doi:10.1371/journal.pone.0184223)
Supplement: S4 Table — (DOCX) [file pone.0184223.s007.docx]

**S4 Table.** Difference in bacterial abundance at the phylum level among the three soil management regimes.

| **Phylum** | **Group** | **Group** | **Sig.** | **Pylum** | **Group** | **Group** | **Sig.** | **Phylum** | **Group** | **Group** | **Sig.** |
| --- | --- | --- | --- | --- | --- | --- | --- | --- | --- | --- | --- |
| Acidobacteria | BF | FW | 0.083 | Firmicutes | BF | FW | 0.077 | Actinobacteria | BF | FW | 0.459 |
|  |  | NF | 0.355 |  |  | NF | 0.284 |  |  | NF | 0.719 |
|  | FW | BF | 0.083 |  | FW | BF | 0.077 |  | FW | BF | 0.459 |
|  |  | NF | 0.349 |  |  | NF | 0.403 |  |  | NF | 0.283 |
|  | NF | BF | 0.355 |  | NF | BF | 0.284 |  | NF | BF | 0.719 |
|  |  | FW | 0.349 |  |  | FW | 0.403 |  |  | FW | 0.283 |
| Bacteroidetes | BF | FW | 0.196 | Chloroflexi | BF | FW | 0.378 | Gemmatimonadetes | BF | FW | 0.666 |
|  |  | NF | 0.189 |  |  | NF | 0.835 |  |  | NF | 0.800 |
|  | FW | BF | 0.196 |  | FW | BF | 0.378 |  | FW | BF | 0.666 |
|  |  | NF | 0.983 |  |  | NF | 0.494 |  |  | NF | 0.856 |
|  | NF | BF | 0.189 |  | NF | BF | 0.835 |  | NF | BF | 0.800 |
|  |  | FW | 0.983 |  |  | FW | 0.494 |  |  | FW | 0.856 |
| Nitrospirae | BF | FW | 0.125 | TM7 | BF | FW | 0.145 | Planctomycetes | BF | FW | 0.150 |
|  |  | NF | 0.609 |  |  | NF | 0.277 |  |  | NF | 0.970 |
|  | FW | BF | 0.125 |  | FW | BF | 0.145 |  | FW | BF | 0.150 |
|  |  | NF | 0.271 |  |  | NF | 0.666 |  |  | NF | 0.141 |
|  | NF | BF | 0.609 |  | NF | BF | 0.277 |  | NF | BF | 0.970 |
|  |  | FW | 0.271 |  |  | FW | 0.666 |  |  | FW | 0.141 |
| Verrucomicrobia | BF | FW | 0.363 | Proteobacteria | BF | FW | 0.369 |  |  |  |  |
|  |  | NF | 0.020* |  |  | NF | 0.872 |  |  |  |  |
|  | FW | BF | 0.363 |  | FW | BF | 0.369 |  |  |  |  |
|  |  | NF | 0.005* |  |  | NF | 0.454 |  |  |  |  |
|  | NF | BF | 0.020* |  | NF | BF | 0.872 |  |  |  |  |
|  |  | FW | 0.005* |  |  | FW | 0.454 |  |  |  |  |

Sig., significance. The difference in community compositions among the four management treatments was carried out with the metastats (http://metastats.cbcb.umd.edu) command of the mothur software (version 1.31.2) and *P* values of < 0.05 were considered signiﬁcant (*). FW, fertilized wheat; NF, natural fallow; BF, bare fallow.
